# Lipedema and the Evolution to Lymphedema With the Progression of Obesity

**DOI:** 10.7759/cureus.11854

**Published:** 2020-12-02

**Authors:** Lívia Maria Pereira de Godoy, Henrique Jose Pereira de Godoy, Paula Pereira de Godoy Capeletto, Maria de Fatima Guerreiro Godoy, Jose Maria Pereira de Godoy

**Affiliations:** 1 General Practice, Clínica Godoy, São José do Rio Preto, BRA; 2 General Practice, Clínica Godoy, Sao Jose do Rio Preto, BRA; 3 Medicine, São José do Rio Preto School of Medicine (FAMERP), São José do Rio Preto, BRA; 4 Cardiology and Cardiovascular Surgery, São José do Rio Preto School of Medicine (FAMERP), Sao Jose do Rio Preto, BRA; 5 Angiology and Vascular Surgery, Clínica Godoy, Sao Jose do Rio Preto, BRA

**Keywords:** lipedema, lymphedema, obesity, bioimpedance

## Abstract

Aim: The aim of the present study was to evaluate the prevalence of subclinical and clinical systemic lymphedema in patients with lipedema and different body mass index (BMI) values.

Method: A cross-sectional study was conducted to determine the prevalence of subclinical systemic lymphedema and clinical lymphedema of the lower limbs detected by bioimpedance (InBody S10 device, Seoul, Korea) in 258 women with clinically diagnosed lipedema. The patients were divided into three groups based on BMI: Group I - BMI below 30 kg/m^2^; Group II - BMI between 30 and 40 kg/m^2^; and Group III - BMI 40 to 50 kg/m^2^.

Results: Fisher's exact test revealed a statistically significant difference between Group I and both Groups II and III (p = 0.0001) regarding the occurrence of lower limb lymphedema.

Conclusion: Patients with lipedema can develop edema even when their weight is within the standards of normality. However, obesity is an aggravating factor, as the prevalence of lipedema increases progressively with the increase in weight.

## Introduction

Lipedema was first described by Allen and Hines in 1940 as a clinical condition associated with the enlargement of the extremities [1]. Individuals with this condition exhibit greater redistribution of fatty tissue to the extremities, which leads to enlargement of the limb [2]. The physiopathological processes are not well defined, but changes in the lymphatic system and fatty tissue are the most cited [2-8]. Polygenic combinations of hormonal, microvascular, and lymphatic disorders may be partially related to the development of lipedema [9]. Some surgical options are reported for select cases, such as liposuction and excisional lipectomy [2].

In one study, the lipedema group had a larger area of adipocytes in comparison to the control group and a similar area to that found in obese controls and obese individuals with lipedema. The number of macrophages increased significantly in the skin and fat in the lipedema group compared to the control group. There was also an increase in dermal vessels in the non-obese lipedema group compared to the control group [10].

The psychological effects of the increase in the volume of the limb and pain are the aspects that most impact the quality of life of these patients [11]. Recent studies describe a novel concept of lymphedema associated with obesity evaluated using bioelectrical impedance analysis. In an initial phase, an increase in intracellular and extracellular liquids is detected in all extremities and the trunk without reaching the stage of clinical lymphedema. This initial stage is denominated subclinical systemic lymphedema. When reading the clinical stage in all extremities and the trunk, the patient has clinical systemic lymphedema [11-14].

The present study aimed to evaluate the prevalence of subclinical and clinical systemic lymphedema in patients with lipedema and different body mass index (BMI) values.

## Materials and methods

Patients and setting

We evaluated 258 women with a clinical diagnosis of lipedema at the Clínica Godoy in 2019.

Design

A cross-sectional study was conducted to determine the prevalence of subclinical systemic lymphedema and clinical lymphedema of the lower limbs detected by bioimpedance (InBody S10 device, Seoul, Korea) in 258 women with clinically diagnosed lipedema.

Inclusion criteria

All women at the clinic for the treatment of stage I and II varicose veins and a diagnosis of associated lipedema.

Exclusion criteria

Women with other causes of clinically diagnosed edema, such as heart failure, kidney failure, and hypoproteinemia.

Development

All patients who met the inclusion criteria were consecutively submitted to bioelectrical impedance analysis for the investigation of subclinical systemic lymphedema, which is characterized by intracellular and extracellular liquids as well as liquids of the lower limb, upper limbs and trunk above normal values. The diagnosis of lower limb lymphedema was based on bioimpedance criteria. The patients were divided into three groups based on BMI: Group I - BMI below 30 kg/m^2^; Group II - BMI between 30 and 40 kg/m^2^; and Group III - BMI 40 to 50 kg/m^2^. Subclinical systemic lymphedema and clinical lower limb lymphedema were investigated in each participant. This study received approval from the Human Research Ethics Committee of the São José do Rio Preto School of Medicine, São Paulo, Brazil (#2.928.922).

## Results

Two hundred fifty-eight consecutive women with lipedema were evaluated using the InBody S10 bioimpedance device: 98 in Group I (BMI less than 30 kg/m^2^), 124 in Group II (BMI between 30 and 40 kg/m^2^), and 36 in Group III (BMI between 40 and 50 kg/m^2^). Among the 98 women in Group I, 16 (16.3%) had subclinical systemic lymphedema (SSL). Moreover, 60 of the 124 women in Group II (48.3%) and 26 of the 36 women in Group III (72.2%) had SSL. Fisher's exact test revealed a statistically significant difference among the groups regarding the occurrence of SSL (p = 0.0001). A significant difference was also found between Groups II and III (p = 0.001).

Clinical lymphedema of the lower limbs was detected in six of the 98 women in Group I (6.1%), 64 of the 124 women in Group II (51.6%), and 28 of the 36 women in Group III (77.8%). Fisher's exact test revealed a statistically significant difference between Group I and both Groups II and III (p = 0.0001) regarding the occurrence of lower limb lymphedema. A significant difference was also found between Groups II and III (p = 0.001).

## Discussion

The present study shows that obesity is an aggravating factor of edema, as edema was progressively frequent with the increase in BMI among women with lipedema. Moreover, approximately 16% of the women with a BMI lower than 30 kg/m^2^ had subclinical systemic lymphedema. This finding suggests that normal fatty tissue in non-obese women with lipedema exhibits important changes associated with the development of edema. However, obesity is an aggravating factor. This is the first study to offer such information.

A previous study found no significant change in the expression of genes associated with inflammation in adipose-derived stem cells (ASCs) in lipedema or differentiated adipocytes [10]. These data suggest that there is no inflammatory process that may cause the edema, at least not in non-obese individuals. Thus, there must be other physiopathological processes.

The vascular changes in microcirculation, especially lymphatic vessels, suggest lymphostasis [4,5,8,15]. A study involving lymphoscintigraphy demonstrated lymphatic alterations in lipedema, suggesting lymphedema resulting from the lymphatic microcirculation [15]. The treatment of these patients using lymphatic drainage results in a reduction of the symptoms and circumference of the limb, which confirms the existence of the lymphatic component [9].

Other conditions associated with edema, such as idiopathic cyclic edema and cellulite, are common in these women. According to a study in the publication phase, 10% have idiopathic cyclic edema. Moreover, cellulite is found in more than 50% of women. Therefore, these are two aggravating factors generally associated with edema [9,16]. Another study detected an increase in the sodium content of adipose tissue in patients with lipedema [17]. Thus, multiple factors are involved in the formation of edema, but all processes lead to an overload of the lymphatic system.

Another important aspect of lipedema is the greater potential for adipogenic differentiation in comparison to patients without this condition [10]. This aspect suggests greater facility in the development of obesity. Thus, the most important advice for these patients is not to gain weight, thereby avoiding the addition of another physiopathological process.

Obesity is the main aggravating factor of edema in these patients, but serious harm can occur in the lymphatic system, leading to overload and the formation of lymphedema. In terms of treatment, the combination of effective lymphatic drainage techniques and prophylactic weight loss to avoid the aggravation of the condition is the best therapeutic approach. 

A perspective to be analyzed in future research would be to have a control group with patients without the presence of lipedema and other types of edema. Also, evaluating therapeutic responses in the short and long term will provide us with greater parameters for the clinical application and treatment of these patients.

## Conclusions

Patients with lipedema can develop edema even when their weight is within the standards of normality. However, obesity is an aggravating factor, as the prevalence of lipedema increases progressively with the increase in weight. Therefore, it is suggested to assess whether weight loss alone reduces this edema.
